# Spatial distribution and determinants of Early sexual initiation in Ethiopia

**DOI:** 10.1186/s12889-024-19057-w

**Published:** 2024-06-07

**Authors:** Shimels Derso Kebede, Natnael Kebede, Mengistu Mera Mihiretu, Ermias Bekele Enyew, Kokeb Ayele, Lakew Asmare, Fekade Demeke Bayou, Mastewal Arfaynie, Agmasie Damtew Walle, Yawkal Tsega, Abel Endawkie

**Affiliations:** 1 Department of Health Informatics, School of Public Health, College of Medicine and Health Science, Wollo University, Dessie, Ethiopia; 2 Department of Health Promotion, School of Public Health, College of Medicine and Health Sciences, Wollo University, Dessie, Ethiopia; 3 Department of Health System and Management, School of Public Health, College of Medicine and Health Sciences, Wollo University, Dessie, Ethiopia; 4 Department of Epidemiology and Biostatistics, School of Public Health, College of Medicine and Health Sciences, Wollo University, Dessie, Ethiopia; 5 Department of Reproductive and Family Health, School of Public Health, College of Medicine and Health Sciences, Wollo University, Dessie, Ethiopia; 6 Department of Health Informatics, School of Public Health, Asrat Woldeyes Health Science Campus, Debre Berhan University, Debre Berhan, Ethiopia

**Keywords:** Early sexual initiation, Sexual health, Spatial distribution, Multilevel logistic regression, Survey, Performance monitoring for action, PMA, Ethiopia

## Abstract

**Introduction:**

: Early sexual initiation has negative health, social, and economic consequences for both women and future generations. The trend of early sexual initiation is increasing globally, leading to higher rates of sexually transmitted diseases and unplanned pregnancies. Ethiopia has been challenged various disasters that makes women vulnerable and position them at heightened risk of early sexual initiation in the last four years. The spatial patterns and factors of early sexual initiation in the post-conflict-post pandemic settings is not well understood. Hence this research aimed at mapping Spatial Patterns and identifying determinant factors in the Post-COVID-Post-Conflict Settings.

**Methods:**

The study was conducted on secondary data from the PMA 2021 cross-sectional survey which conducted nationally from November 2021 to January 2022 which is in the post pandemic and post-war period. Total weighted sample of 6,036 reproductive age women were included in the analysis. ArcGIS Pro and SaTScan software were used to handle spatial analysis. Multilevel logistic regression model was used to estimate the effects of independent variables on early sexual initiation at individual and community level factors. Adjusted odds ratio with the 95% confidence interval was reported to declare the strength and statistical significance of the association.

**Result:**

The spatial distribution of early sexual initiation was clustered in Ethiopia with a global Moran’s I index value of 0.09 and Z-score 6.01 (p-value < 0.001).Significant hotspots were detected in East Gojjam zone of Amhara region, Bale, Arsi, West Hararge, East Wellega and Horo Gudru Wellega zones of Oromia region. The odds of having early sexual initiation was higher in women with primary education (AOR = 1.23, 95%CI: 1.03, 1.47), secondary or above education (AOR = 4.36, 95%CI: 3.49, 5.44), Women aged 26 to 25 (AOR = 1.91, 95%CI: 1.61, 2.26), women aged 36 to 49(AOR = 1.51, 95%CI: 1.24, 1.84). However, there was a significant lower likelihood of early sexual initiation in rural resident women (AOR = 0.53, 95%CI: 0.35, 0.81) and women living in 5 to 7 family size (AOR = 0.79, 95%CI: 0.68, 0.92), and more than 7 members (AOR = 0.63, 95%CI: 0.49, 0.81).

**Conclusions:**

The spatial distribution of early sexual initiation was clustered in Ethiopia. Interventions should be taken to eliminate the observed variation by mobilizing resources to high-risk areas. Policies and interventions targeted to this problem may also take the identified associated factors into account for better results.

## Introduction

Early sexual initiation was defined as the experience of having sexual intercourse before the age of 18 years [1]. But women before the age of 18 are considered children and they couldn’t decide concerning marriage and/or consensual sexual relationship, as the Universal Declaration of Human Rights proclamation [2]. Early sexual initiation has negative health, social, and economic consequences for both women and future generations. It raises the risk of sexually transmitted diseases such as HIV and AIDS, as well as the risks of unwanted pregnancy [3, 4]. As a result of cervical immaturity, early sexual debuts are also associated with an increased risk of infection by the Human Papillomavirus (HPV), which leads to cervical cancer [5]. Further, early sexual initiators are less likely to complete their schooling and attending higher education due to the possibility of becoming pregnant, which limits their prospects for social and vocational advancement in the future [6, 7]. A growing body of evidence indicates that young people who engage in sexual activity at an early age are more likely to engage in risky sexual behaviours, including multiple sexual partners and improper or inconsistent condom use [8–10]. Consequently, this act increases unsafe abortions, childbirth at a young age, and psychosocial problems. Due to the lack of knowledge about sexual health and the risks associated with it, these problems are the greatest threats to the health and well-being of the youth population in general. It is common for young people to lack the information and guidance they need to make informed decisions about their sexual behaviour, which can lead to risky behaviours and serious consequences [11, 12]. Also, it led to a higher rate of school dropouts, poor school performances, stigma, and discrimination [13–15]. As a result, it adversely affects an individual’s social and economic status in their childhood [16]. This, in turn, can lead to isolation, poverty and other negative consequences later in life.

The trend of early sexual initiation is increasing globally, leading to higher rates of sexually transmitted diseases and unplanned pregnancies [17]. The prevalence of early sexual initiation globally varies by region, overall, approximately 14.2% of adolescents aged 12–15 years have early sexual initiation, with the highest prevalence in the Americas region and lowest in the South-east Asia region [18, 19]. Findings also highlight a significant burden of early sexual initiation in Africa, with, the pooled prevalence of 21.14% among female youth in Eastern Africa [20], to prevalence ranged from 14.4 to 40.1% among adolescent girls and young women in sub-Saharan Africa [18].

The prevalence of early sexual initiation in Ethiopia is also significantly high, with variation across different populations. In 2016, the national prevalence was 66.95% among reproductive-age women aged 15 to 49, and the proportion of early sexual initiation was 17.9% among college students in Southwest Ethiopia [20, 21]. Furthermore, the prevalence of early sexual initiation was 38.4% among young girls aged 15 to 24 nationally [22]. Ethiopia have been challenged through various natural and man-made disaster including COVID-19 pandemic and armed-conflicts in different parts of the country over the past four years. In such post-conflict and post-pandemic settings, individuals, especially young women and girls, are at heightened risk of early sexual initiation. In post-conflict settings, the breakdown of traditional support systems and social norms, displacement, and trauma can make women more vulnerable to exploitation. The COVID-19 pandemic has worsened vulnerabilities by restricting movement, causing economic strain, and disrupting essential services. Additionally, the closure of schools and support programs has limited access to sexual and reproductive health services, leaving them more susceptible to exploitation. Various researches have been done on the prevalence and factors associated with early sexual initiation in Ethiopia. Age, residence, educational status, parent-youth discussion, using addictive substances and religion were determinant factors identified [23–29]. Furthermore, the prevalence and spatial distribution of early sexual initiation was assessed based on data from Ethiopian demographic and health survey (EDHS) 2016 and previous surveys, which is before the COVID outbreak and pre-conflict in Ethiopia [1, 30]. However, the spatial distribution and determinant factors of early sexual initiation in post-conflict settings is not investigated. Spatial analyses, including hot spot and spatial scan analyses, offer valuable insights into the spatial distribution and determinants of early sexual initiation.

While previous studies have not explored these spatial dynamics, the unique socio-economic and environmental factors in such contexts warrant a spatial perspective. By identifying spatial clusters and potential determinants of ESI, these analyses can inform targeted interventions and resource allocation to areas with the highest need. Incorporating spatial perspectives into our analysis will enhance our understanding of ESI in post-conflict settings and support the development of effective strategies to address this critical issue. Hence this research aimed at mapping spatial patterns and identifying determinant factors in the Post-COVID-Post-Conflict settings. By uncovering patterns and influences in post-war environments, this research contributes crucial insights for public health initiatives by informing strategies and contextually appropriate interventions to improve public health outcomes. These insights support evidence-based decision-making in public health policy and programming, ultimately improving health outcomes for affected populations. Additionally, understanding the spatial dynamics aids in planning reproductive health services, allocating resources effectively, and tailoring programs to the specific needs of communities.

## Methods

### Study design and period

The study was conducted on secondary data from cross-sectional the 2021 Performance Monitoring for Action Ethiopia (PMA Ethiopia) household and female survey. PMA Ethiopia 2021 cross-sectional survey used a two-stage cluster design. All women age 15–49-years old in the selected households are eligible for the cross sectional survey. The data collection was conducted from November 2021 to January 2022 [31].

### Source and study population

Reproductive age women (aged 15–49) in Ethiopia was the source population, and reproductive age women from selected enumeration areas were considered a study population. Hence, a total weighted sample of 6,036 reproductive age women were included in the analysis.

### Study variables

#### Dependent variable

Early sexual initiation was the dependent variable which was dichotomized into two categories such as ‘yes’ coded 1 if a woman started sexual intercourse before the age of 18 years and ‘no’ coded 0 if not.

### Independent variables

The independent variables for early sexual initiation included in the analysis were individual level factors such as women’s education level (highest level of school attended), family size (the number of household members), women age, phone ownership (women’s own a phone? ), and community level variables such as place of residence (women live in urban or rural community),, region (administrative region women live), community education level(whether the community that a woman live in have higher or lower level of education), and community level poverty (whether community level poverty is high or low).

### Data processing and analysis

Descriptive statistics such as frequencies and percentages were computed to better understand the characteristics of respondents and multilevel logistic regression was fitted to assess the relationship between early sexual initiation and the independent variables. The data processing and model development was handled through Stata version 17 and further spatial analysis was done using ArcGIS pro 2.8 & SaTScan^™^ 10.0.2. PMAET 2021 Cross-sectional survey was conducted on 243 enumeration areas (EA) selected from the master sample frame of the Ethiopian Central Statistical Agency, with 35 randomly selected households within each enumeration area [31]. The centroid in each EA is found using GPS coordinate data from all listed households within the EA. These enumeration areas GPS data are recorded as geographic coordinates (degrees in latitude and longitude) and used for spatial analysis by linking them with the survey data by using the “EA_ID” variable/column.Spatial analysis.

### Spatial autocorrelation analysis

Spatial autocorrelation (Global Moran’s I) statistic was used to assess whether early sexual initiation is clustered, dispersed, or randomly distributed in Ethiopia. Spatial autocorrelation analysis is a method used to analyze the correlation between spatially adjacent events based on their attribute values. Moran’s I is a statistic used to quantify spatial autocorrelation. Moran’s I value closer to − 1 indicates that the event of interest is dispersed, whereas closer to + 1 indicates clustering effect, and a random spatial pattern if closer to 0 [32]. A statistically significant Moran’s I value (*p* < 0.05) demonstrates the presence of non-random spatial pattern of early sexual initiation.

### Hot spot analysis

Hotspot analysis was done through Gettis-ord Gi* statistics, which could identify hot spot areas with computing z-score to confirm the statistical significance of clustering at p-value < 0.05 with 95% confidence interval [33–35]. If the z-score is between − 1.96 and + 1.96, the p-value would be larger than 0.05, and, the spatial pattern could be due to chance whereas the observed spatial pattern is significant if the z-score falls outside the range [36]. Statistical output with a high Gi* value suggests a hotspot, indicating a high proportion of early sexual initiation in the area, whereas a low Gi* value indicates a cold spot, indicating a low proportion of early sexual initiation.

### Spatial scan analysis

Pure spatial scan statistical analysis was applied to test for the presence of statistically significant spatial clusters of early sexual initiation. Spatial scan analysis strengthens the findings detected by Gettis-Ord analysis and allows the study to report confirming findings [34]. The Bernoulli model was fitted by considering a woman who had early sexual initiation as a case and a woman who didn’t have sex before 18 years of age as a control. Potential clusters of early sexual initiation were ranked based on their log likelihood ratio (LLR) test with corresponding p-values and relative risk. Clusters with a p-value < 0.05 were considered significant hotspot areas, with the first-ranked cluster as the primary cluster and the rest as secondary clusters.

### Spatial interpolation

Spatial interpolation technique through kriging tool was used to predict early sexual initiation at originally non-sampled enumeration areas during the survey. For choosing the optimal interpolation technique, deterministic and geostatistical interpolation techniques were compared with their mean prediction error (MPE) and root mean squared prediction error (RMSPE) after predicting early sexual initiation in originally un-sampled enumeration areas.

### Multilevel analysis

Multilevel logistic regression model was used to estimate the effects of independent variables on early sexual initiation at individual and community level factors. A total of four multilevel models were fitted. First, the null model (Model I) without independent factors was fitted to estimate the random intercept at cluster level and the variation in the odds of early sexual initiation between communities. Second, model II was fitted on individual-level factors. Third, model III was constructed on community level factors. Finally, model IV was fitted on both individual and community level variables. These models were compared and selected by their deviance. The model with the lowest deviance was the best models and fitted to estimate the association between independent factors and early sexual initiation in Ethiopia. The random effect measures used to measure the variation were estimated using the Median Odds Ratio (MOR), Intra-class Correlation Coefficient (ICC), and Proportional Change in Variance (PCV). Model eligibility was assessed by calculating the intra-class correlation coefficient (ICC), and a model with an ICC greater than 10% considered eligible for multilevel analysis. The ICC for this study was 26.8%, an indication for individuals nested within the community.

Finally, bi variable multilevel logistic regression analysis was performed and all independent variables that were statistically significant at p value < 0.2 were included in the multivariable logistic regression model. In multivariable multilevel logistic regression models, variables with p value < 0.05 were considered significant and their strength of association with the dependent variable was reported through adjusted odds ratio (AOR) at 95% confidence interval (CI).

## Result

### Characteristics of the study population

Among a total weighted sample of 6035 reproductive age women, 2443 (40.49%) of them were not having any formal education at all, and about 2,321 (38.45%) and 1,271(21.06%) have primary, and secondary or above educational level respectively (Table 1). More than half of women in this study were from a community with a higher proportion of poverty (56.27%) and community with lower proportion of education (55.82%). The majority of the respondents were also (71.19%) rural residents. Regarding the regional distribution of the respondents, majority of women were from Oromia (43.96%) followed by 1,458 (24.15) respondents from Amhara region and Southern nations and nationalities and peoples (SNNP) region (14.80%).

**Table 1:** Characteristics of the study population for early sexual initiation in Ethiopia, PMA 2021

Variables	Categories	Weighted frequency	Percentage
education level	No education	2,443	40.49
	Primary	2,321	38.45
	Secondary or above	1,271	21.06
family size	1 to 4	2,914	48.28
	5 to 7	2,478	41.06
	> 7	643	10.66
Have Phone	No	2,883	49.93
	Yes	2,890	50.07
age group	15 to 15	1,855	30.74
	26 to 35	2,312	38.30
	36 to 49	1,868	30.96
Place of Residence	urban	1,739	28.81
	rural	4,296	71.19
Region	Tigray^**†**^	0	0
	Afar	81	1.34
	Amhara	1,458	24.15
	Oromia	2,653	43.96
	Somali	239	3.97
	Benishangul-Gumuz	69	1.14
	SNNP	893	14.80
	Gambella	29	0.47
	Harari	21	0.35
	Addis Ababa	343	5.69
	Dire Dawa	31	0.51
	Sidama	219	3.62
community education	low	3,369	55.82
	high	2,666	44.18
Community level poverty	low	2,639	43.73
	high	3,396	56.27

**†** Tigray was not incorporated in PMA 2021 due to security reasons

### Spatial analysis result

#### Spatial autocorrelation

**Fig. 1:**
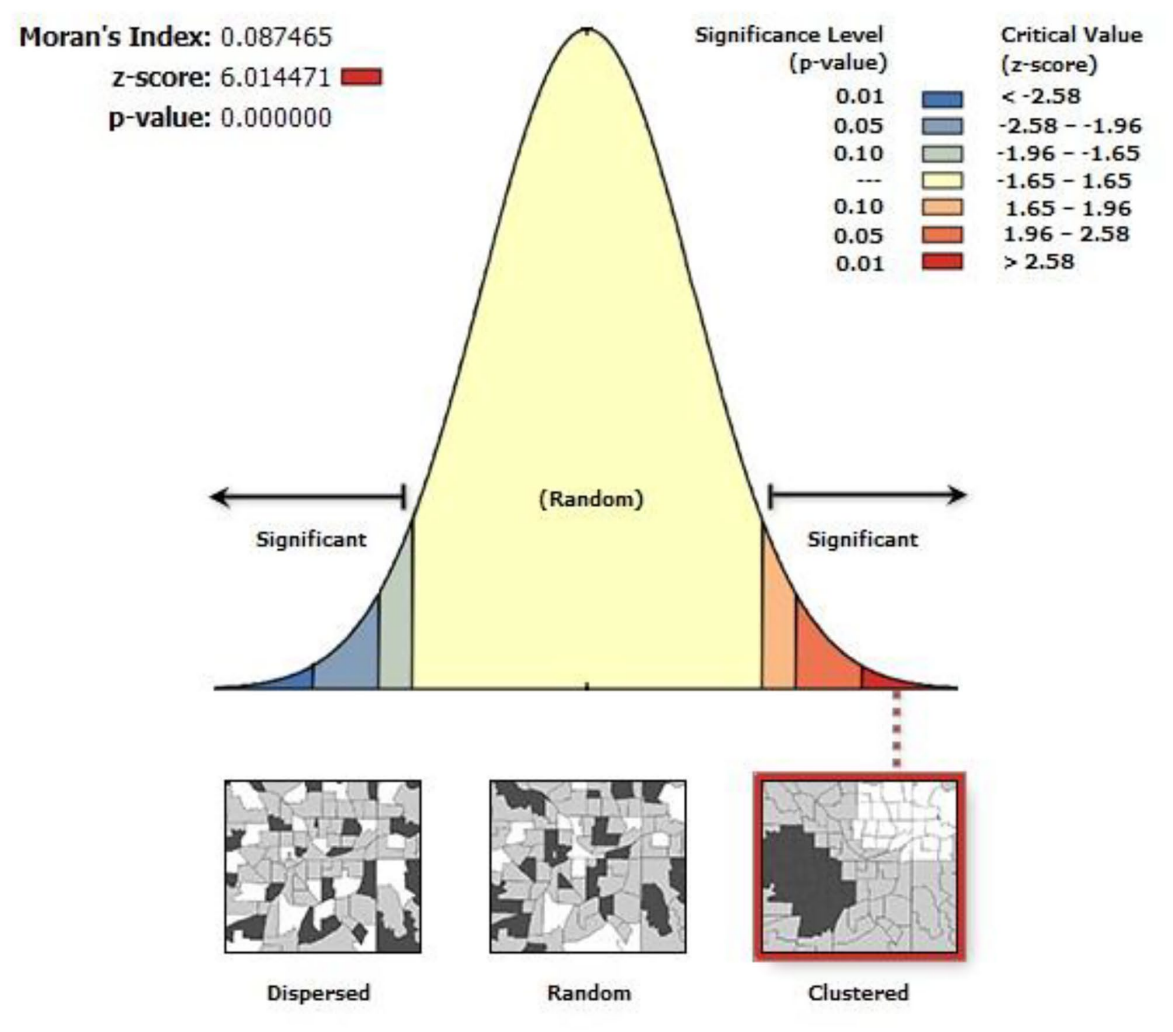
Spatial autocorrelation of early sexual initiation in Ethiopia

The spatial distribution of early sexual initiation was clustered in Ethiopia with a global Moran’s I index value of 0.09 and Z-score 6.01 (p-value < 0.001), a statistically significant clustering effect (Fig. 1). Given the z-score of 6.01, there is a less than 1% likelihood that this clustered pattern of early sexual initiation could be the result of random chance.

### Hotspot (Getis-Ord Gi*) analysis

**Fig. 2:**
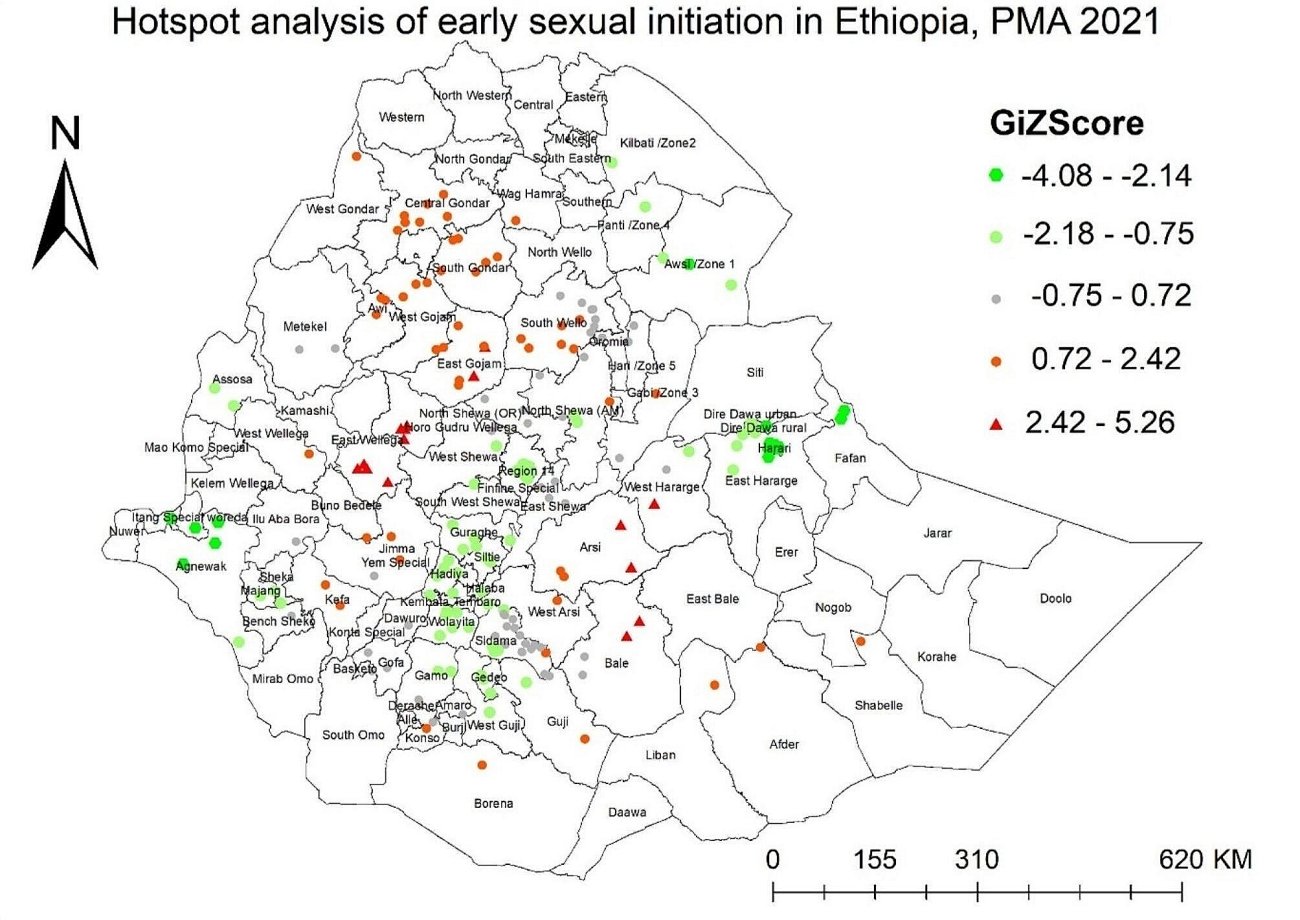
Hotspot and cold spots of early sexual initiation in Ethiopia

The highest risky areas for early sexual initiation were detected in East Gojjam zone of Amhara region, Bale, Arsi, West Hararge, East Wellega and Horo Gudru Wellega zones of Oromia region with a Gi Z score of ranging 2.42–5.26 indicating significant hotspots at 95% confidence interval (Fig. 2). North and South Gondar zones of Amhara region, West Wollega, and Horo Guduru zones of Oromia region, Keffa and Gamo Gofa zones of SNNP, and Nogob, Jarar, and north western part of Shabelle zones in Somali region .

West Gondar, South Gondar, Central Gondar, Awi, West Gojjam, and South Wollo zones of Amhara region, West Wellega, Jimma, West Arsi, Guji, and Borena zones of Oromia region, Zone 3 of Afar region, Liben, Afder, and Shabele zones of Somali region, keffa zone of South West Ethiopia region, Konso zone in SNNP, and south northern border of Sidama region were also hotspot areas with a Gi Z score range of 0.72 to 2.42, means significant hotspots at 90% confidence interval. Whereas Harari and Dire Dawa, Zone 1, Zone 2, and Zone 4 of Afar region, Fafan zone of Somali region, Itang special woreda and Agnewak zone of Gambela region, Assosa zone of Benshangul Gumuz region, Guraghe, Silte, Hadiya, Halaba, Tembaro, Kambata, welaita, and Gamo zones of SNNP regions, and Sheka zone South West Ethiopia region were cold spot areas for early sexual initiation (Fig. 2).

### Spatial scan analysis

**Fig. 3:**
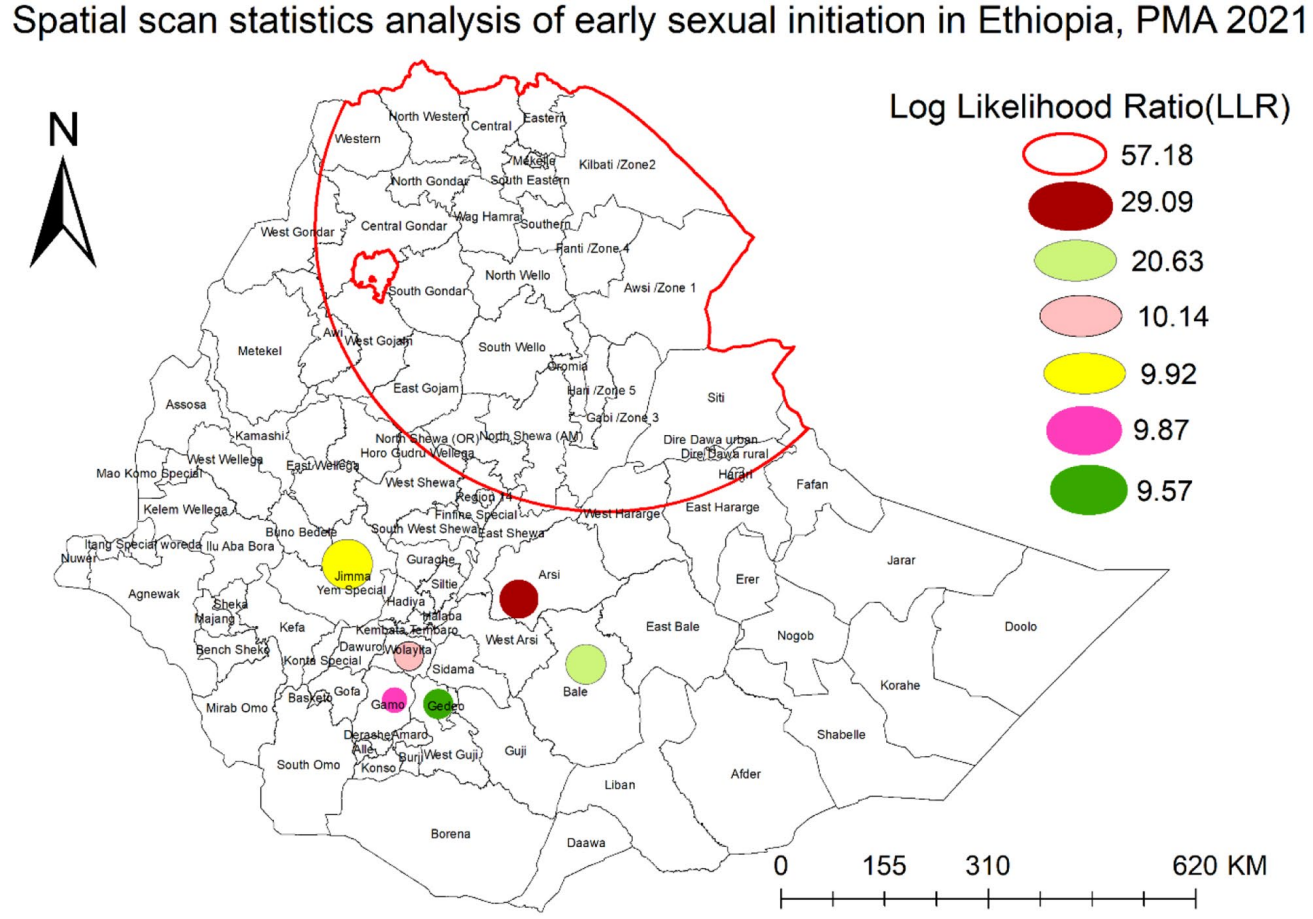
Spatial scan statistics analysis of early sexual initiation in Ethiopia

Bernoulli based purely spatial analysis at a maximum spatial cluster size of ≤ 50% of the population at risk detected one primary cluster and six secondary clusters (Table 2; Fig. 3). The primary cluster for was centered at 12.580526 N, 40.396862 E with a 421.73 Km radius and has a relative risk of 1.20 (LLR = 57.18, p-value < 0.001). Afar, Tigray. Amhara, Harari, Dire Dawa, and West Shewa, West Harerge, and East Harerge zones of Oromia, Siti and Fafan zones in Somali region were located in this cluster. For women who lived in enumeration areas within this areas, the risk of early sexual initiation increases by 20% relative to women outside this cluster.

The second most likely cluster was detected in Arsi zone of Oromia region, centered at 7.609242 N, 39.246954 E) with a 10.00 km radius and had a relative risk of 1.33 (LLR = 29.09, p-value < 0.001). Women in this cluster had 33% more risk for early sexual initiation compared to women outside this spatial window.

Other significant clusters were also detected in Bale and Jimma zones of Oromia region, and Wolayita, Gamo, and Gedio Zones of SNNP region.

**Table 2:** Summary of satscan result of early sexual initiation in Ethiopia

Detected Cluster	Coordinate/Radius	Population	Cases	RR	LLR	*P*-value
Primary cluster	(12.580526 N, 40.396862 E) / 421.73 km	2021	1611	1.20	57.18	< 0.001
Secondary cluster 1	(7.609242 N, 39.246954 E) / 10.00 km	169	159	1.33	29.09	< 0.001
Secondary cluster 2	(6.732709 N, 40.148105 E) / 29.77 km	113	107	1.34	20.63	< 0.001
Secondary cluster 3	(6.844311 N, 37.767732 E) / 21.72 km	49	47	1.35	10.14	0.006
Secondary cluster 4	(8.078890 N, 36.940035 E) / 37.34 km	118	104	1.24	9.92	0.01
Secondary cluster 5	(6.249460 N, 37.573700 E) / 0 km	29	29	1.41	9.87	0.01
Secondary cluster 6	(6.198869 N, 38.162974 E) / 0 km	67	62	1.30	9.57	0.01

### Interpolation

**Fig. 4:**
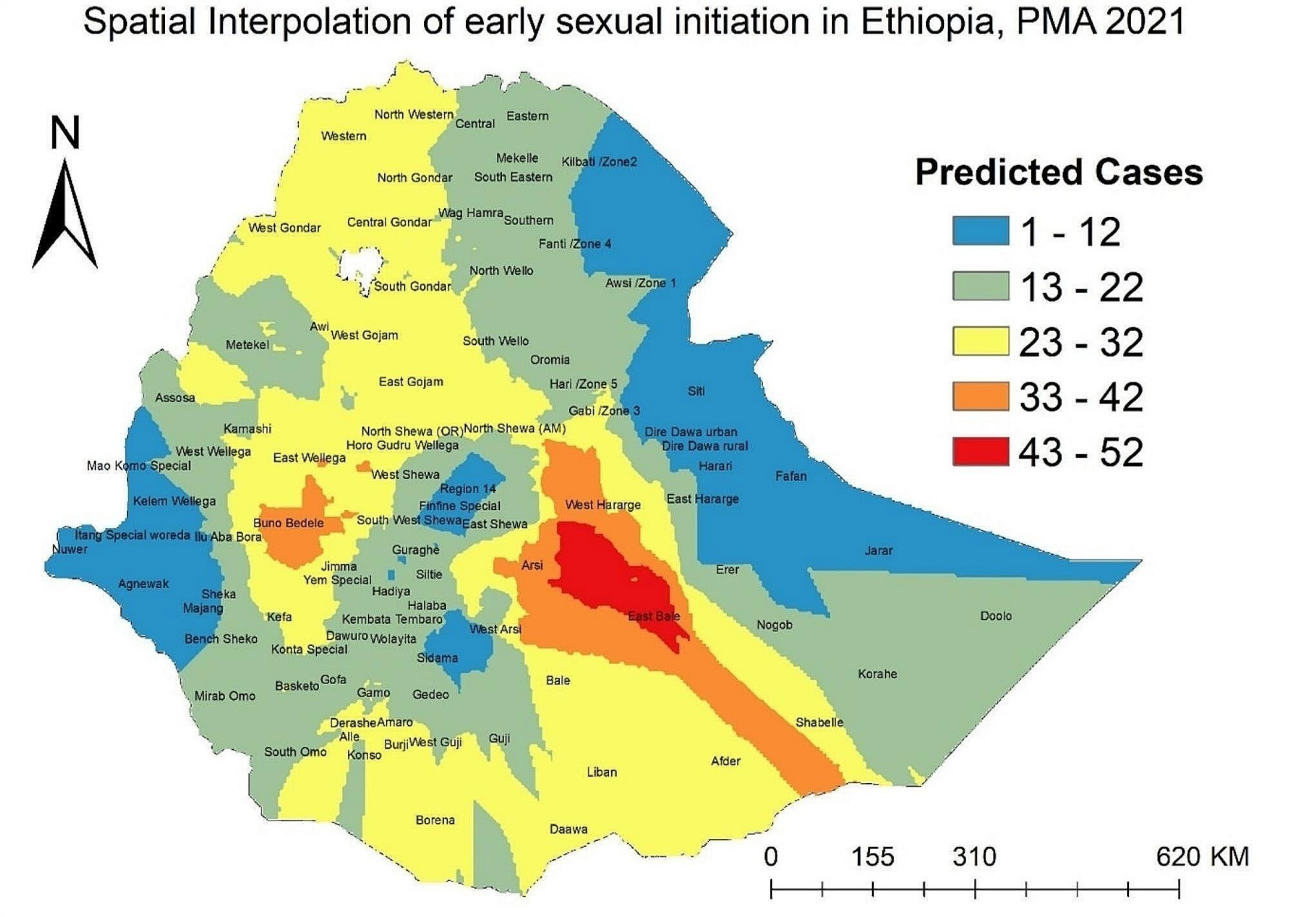
Spatial interpolation of early sexual initiation in Ethiopia

The comparison of different spatial techniques showed that ordinary kriging was the best model to predict early sexual initiation with a mean prediction error of -0.031 and Root Mean Squared Prediction Error (RMSPE) of 13.44 (Table 3). Accordingly, ordinary kriging interpolation result indicated that West and Eastern Hararge, eastern portion of Arsi, northern part of Bale zone and south eastern enumeration areas in East Bale zone had the highest distribution of early sexual initiation. Bunno Bedele, Arsi, West Harerge, East Wellega of Oromia region, and some woredas of Shabele and Afder zones of Somali region also had high distribution of early sexual initiation (Fig. 4).

**Table 3:** Result for comparison of interpolation techniques to predict early sexual initiation

Model	Mean prediction error	RMSPE
IDW	-1.42	13.93
Ordinary kriging	-0.03	13.44
EBK/Empirical Bayesian Kriging	-0.47	13.49

### Multilevel result

#### Random effect (measures of variation) and model fit statistics

The results of the null model revealed that variability between clusters accounted for 26.8% of the total variation in early sexual initiation (ICC = 0.268), and women from clusters with higher proportion early sexual initiation was 2. 83 times more likely to have early sexual initiation than, women from a cluster with lower proportion early sexual initiation (MOR = 2.83). As model complexity increased from null model to full model, the values of Akaike’s information criterion(AIC), Bayesian information criterion(BIC), and Deviance decreased, indicating that the final model was best model with AIC and Deviance values of 5881.72 and 5837.32, respectively. Furthermore, the PCV value in the final model was 56.7% indicating that the variation of early sexual initiation among reproductive women in Ethiopia was explained by the final model (Table 4).

**Table 4:** Parameters and model fit statistics for multilevel regression analysis models

Parameters	Null model (Model I)	Model II	Model III	Model IV
Random effect				
Community variance	1.20 [0.93, 1.55]	0.75(0.57, 1.01)	0.59 (0.44, 0.79)	0.52(0.38,0.70)
ICC	26.8%	18.7%	15.2%	13.6%
MOR	2.83[2.5, 3.26]	2.28(2.05, 2.60)	2.07(1.88, 2.33)	1.98(1.80,2.21)
PCV	1	37.5%	50.83%	56.7%
Model comparison				
AIC	6539.33	5920.21	6445.99	5881.72
BIC	6552.74	5980.11	6546.55	6028.14
LLR	-3267.66	-2951.11	-3208	-2918.86
Deviance	6535.32	5902.22	6416	**5837.32**

NB: **AIC**: Akaike’s information criterion, **BIC**: Bayesian information criterion, **LLR**: Log likelihood, **MOR**: Median Odd Ratio, **ICC**: Intra-class Correlation Coefficient and **PCV** (Proportional Change in Variance)

### Determinants of early sexual initiation

In the full model adjusted for individual and community-level factors, women education, family size, women’s age, place of residence, and region were significantly associated with early sexual initiation (Table 5).

The odds of having early sexual initiation was 23% higher in women with primary education (AOR = 1.23, 95%CI: 1.03, 1.47), and 4.36 times higher in secondary and above education (AOR = 4.36, 95%CI: 3.49, 5.44), compared with women who had no formal education. The odds of having early sexual initiation among women in 5 to 7 family size, and family size of more than 7 members were decreased by 21% (AOR = 0.79, 95%CI: 0.68, 0.92) and 37%(AOR = 0.63, 95%CI: 0.49, 0.81), respectively. Women aged 26 to 25 have 91%( AOR = 1.91, 95%CI: 1.61, 2.26) higher odds of having early sexual initiation than their counterparts. Similarly, women aged 36 to 49 have 51%( AOR = 1.51, 95%CI: 1.24, 1.84) higher odds of early sexual initiation. Compared to urban women, the odds of having early sexual initiation was 47% lower in rural resident women (AOR = 0.53, 95%CI: 0.35, 0.81). The odds of having early sexual initiation among women who live in Amhara region and Gambela region were decreased by 66% [AOR = 0.34, 95%CI: 0.17, 0.71] and 74% (AOR = 0.26, 95%CI: 0.07, 0.95) respectively, compared to those who live in Afar region.

**Table 5:** Multilevel analysis of factors associated with early sexual initiation among reproductive age women in Ethiopia, PMA 2021

Variables	Categories	Null model	Model I AOR (95%CI)	Model II AOR (95%CI)	Model III AOR (95%CI)
education level	No education		Ref		
	Primary		1.26(1.06, 1.51)*		1.23 (1.03, 1.47)*
	secondary and above		4.63 (3.73, 5.75)*		4.36 (3.49, 5.44)*
family size	1 to 4		Ref		
	5 to 7		0.78 (0.67, 0.91)*		0.79 (0.68, 0.92)*
	> 7		0.63 (0.49, 0.81)*		0.63 (0.49, 0.81)*
Have Phone	No		Ref		
	Yes		1.22 (1.03, 1.43)*		1.12 (0.95, 1.32)
age group	15 to 15		Ref		
	26 to 35		1.97 (1.66, 2.32)*		1.91 (1.61, 2.26)*
	36 to 49		1.54 (1.27, 1.88)*		1.51 (1.24, 1.84)*
Place of Residence	Urban			ref	
	Rural			0.39 (0.25,0.60)*	0.53 (0.35, 0.81)*
region	Afar			Ref	
	Amhara			0.43 (0.22,0.89)*	0.34 (0.17,0.71)*
	Oromia			0.65 (0.32,1.33)	0.61 (0.29,1.25)
	Somali			1.49 (0.58,3.84)	1.52 (0.60, 3.85)
	Benishangul-Gumuz			0.76 (0.27,2.14)	0.51 (0.18, 1.48)
	SNNP			0.84(0.40, 0.78)*	0.79( 0.38, 1.68)
	Gambela			0.35 (0.97, 1.25)	0.26 (0.07, 0.95)*
	Harari			0.76 (0.21, 2.76)	0.73 (0.19, 2.72)
	Addis Ababa			1.50 (0.65, 3.43)	1.13( 0.49, 2.59)
	Dire Dawa			1.10 (0.35, 3.44)	1.08 (0.33, 3.52)
	Sidama			0.68 (0.29, 1.60)	0.61 (0.26, 1.44)
community education	Low			Ref	
	High			1.55(1.10, 2.18)*	1.16( 0.83, 1.62)
Community level poverty	Low			Ref	
	High			1.10 (0.73, 1.65)	1.31 (0.89, 1.95)

## Discussion

This study was conducted to determine the spatial distribution of early sexual initiation in Ethiopia in the post conflict setting based on the 2021 PMA survey. Accordingly, the spatial distribution of early sexual initiation was clustered in Ethiopia as of the result of spatial autocorrelation analysis. The finding presented in this study was consistent with previous studies that examined the spatial distribution of early sexual initiation among women in Ethiopia based on 2016 and previous survey of EDHS which reported non-random distribution [1]. In the post conflict era, new hotspots were identified in East and West Wellega zones of Oromia region, and in enumeration areas located across borders of Somali and Oromia regions. Furthermore, even though there is slight reduction of the burden in Amhara region, it is still a high risk area of early age sexual initiation. This might be due to the reason that these regions have faced ongoing political instability and lack of access to comprehensive health services and education. These factors contribute to the vulnerability of young people in these areas, making them more susceptible to engaging in risky behaviors such as early sexual initiation. The identification of hotspots for early sexual initiation in conflict-affected regions underscores the multifaceted impact of conflict on social dynamics and health outcomes. It is obvious that war and conflict have profound and far-reaching consequences when it comes to the occurrence of forced sexual acts and the initiation of sexual activities at an early age [37, 38]. Particularly in the midst of armed conflicts, sexual violence is frequently employed as a strategic tool, with a specific focus on women and girls as vulnerable targets of such heinous acts [39]. Furthermore, pandemics like COVID-19 have been associated with various impacts on children, including potential influences on involving into sexual intercourse in their early age [40, 41]. This highlights the far-reaching and pervasive nature of sexual violence and early-puberty during times of armed conflict and public health emergencies such as pandemics. Despite that, efforts to prevent the emergence of new hotspots should be intensified in areas where the occurrence of such incidents is currently low.

In multilevel analysis, findings suggest that women’s education, family size, women’s age, and place of residence found to be significant determinants of early sexual initiation. The odds of having early sexual initiation were higher in women with education level primary and secondary or above compared with women who had no formal education. This result was in line with a study conducted in Nigeria [42]. The reason behind this could be that most educated women come from urban areas where social media and other pressures to date young are common. Additionally, educated women may have greater exposure to information about sexuality and relationships, which could influence their decision to engage in early sexual initiation. Furthermore, societal expectations and norms in urban areas might place a higher emphasis on exploring one’s sexuality at a younger age, leading to increased rates of early sexual initiation among educated women. For example, a study conducted by Smith et al. found that 80% of educated women surveyed in urban areas reported feeling pressure from social media to engage in early sexual initiation [43]. While increasing access to education, especially for girls, is crucial for empowering individuals and communities, it alone may not suffice to effectively mitigate the issue of early age sexual initiation. Education undoubtedly equips individuals with knowledge and skills, including information about sexual and reproductive health, which can contribute to informed decision-making and potentially delay sexual debut. So, to comprehensively address the multifaceted factors influencing early sexual initiation, special attention and targeted interventions are essential. By adopting a holistic approach that combines education with targeted interventions and community engagement, policymakers and stakeholders can better address the complexities surrounding early age sexual initiation and contribute to the overall well-being of women. The odds of having early sexual initiation were lower as family size grows. This might be due to larger families may provide a more supportive and protective environment for adolescents, reducing their likelihood of engaging in early sexual activity. Furthermore, larger families offer adolescents increased social support, guidance, advice, and emotional support, as well as closer sibling relationships, providing a sense of companionship and protection.

The odd of having early sexual initiation was lower in rural resident women relative to their urban counterparts. This finding was supported by other studies in Ethiopia [20] and Ghana [44]. This could be explained by the possibility of urban women exposure to drugs, alcohol, and pornographic content, which is rare in the rural community. Activities such as alcohol drinking, chewing chat, smoking cigarette, and viewing pornographic materials have been found to be significantly associated with early sexual initiation among adolescents [21, 45, 46]. In line with findings reported in Ethiopia [22] and Nigeria [47], being older age was associated with higher odds of early age sexual intercourse when compared with younger age counterparts. The possible justification could be the earlier generation women were more likely to engage in early marriage and other harmful traditional practices, which are dropped significantly nowadays. This explanation was supported by a study that reported a significant decline of child marriages in Ethiopia over the past ten years [48].

By connecting multilevel model and spatial analyses, efforts have been made to bridge these analyses to provide a coherent interpretation of the findings. Specifically, significant variables identified in the multilevel model have been assisted with spatial clusters or hotspots identified through spatial analysis, enriching the understanding of underlying patterns. This integrated approach allows for a more comprehensive interpretation of the results, facilitating insights into both individual-level predictors and their spatial distribution. Furthermore, by synthesizing these findings, better informing policy and practice, particularly in targeting interventions to geographic areas where individual-level risk factors are concentrated, as revealed by spatial analysis, is possible.

### Strength and limitation of the study

The use of advanced statistical models that consider the hierarchical nature of the research design and take individual and community-level predictors into account is strength of this study, which also improves the quality of the study. Furthermore, the study employed geospatial analysis techniques to examine the spatial distribution of early sexual initiation, allowing for a more comprehensive understanding of its geospatial distributions and potential hotspots. This not only adds depth to the research but also provides valuable insights for targeted interventions and resource allocation.

However, it is important to acknowledge that the exclusion of data from the Tigray region due to unavailability of data and the absence of key variables may limit the generalizability and comprehensiveness of the findings. Therefore, future researchers should consider including data from Tigray and obtaining additional variables to provide a more comprehensive understanding of the topic.

## Conclusion

The spatial distribution of early sexual initiation was clustered in Ethiopia with hotspots in in East Gojjam zone of Amhara region, Bale, Arsi, West Hararge, East Wellega and Horo Gudru Wellega zones of Oromia region. Ordinary kriging interpolation also predicted higher frequency of early sexual initiation in West and Eastern Hararge, eastern portion of Arsi, northern part of Bale zone and south eastern enumeration areas in East Bale zone. The identification and prediction of such high-risk areas could provide useful information to decision-makers targeted at reduction of the problem. Hence, interventions should be taken to eliminate the observed variation by mobilizing resources to high-risk areas while strengthen the efforts to keep the burden lower at identified cold spots.

The results also highlighted a significant association between early sexual initiation and factors such as women education, family size, women’s age, place of residence, and region. Urban resident women, women having higher level of education, and women live in lower family size have a higher odds of engaging in early sexual debut. By prioritizing these factors, policymakers and stakeholders can develop targeted interventions tailored to the needs of these groups, thus effectively reducing the burden of early sexual initiation in the nation.

## Data Availability

The original data is available from PMA website (https://www.pmadata.org).

## Abbreviations

AIC: Akaike’s information criterion
AOR: Adjusted odds ratio
BIC: Bayesian information criterion
CI: Confidence interval
EDHS: Ethiopian demographic and health survey
HPV: Human Papillomavirus
ICC: Intra-class correlation coefficient
LLR: Log likelihood ratio
MOR: Median Odds Ratio
MPE: Mean prediction error
PCV: Proportional Change in Variance
PMA: Performance Monitoring for Action
RMSPE: Root mean squared prediction error
SNNP: Southern nations and nationalities and peoples
